# Cutoffs of different body measurement indexes of central obesity in patients with type 2 diabetes

**DOI:** 10.1038/s41598-024-52645-9

**Published:** 2024-01-25

**Authors:** Ai Luo, Zheng Tang, XiaoJia Xu, Chao Li, Die Zhou, Dong Xiao, Yongjie Lu, Rutao Liang, Guifen Guan, Wangen Li, Zhuoqing Hu

**Affiliations:** 1 Department of Endocrinology, The Second Affiliated Hospital of Guangzhou Medical University, Guangzhou, 510220 China; 2 Guangdong Medical University, Zhanjiang, 524000 China; 3 Department of Laboratory, The Second Affiliated Hospital of Guangzhou Medical University, Guangzhou, 510220 China; 4 School of Public Health, Guangzhou Medical University, Guangzhou, 510220 China

**Keywords:** Endocrinology, Endocrine system and metabolic diseases

## Abstract

Few research discuss whether the body measurement indexs of obesity in general populations is applicable to patients with type 2 diabetes. We explore the optimal cutoffs of visceral fat area (VFA) and subcutaneous fat area (SFA) in the diagnosis of central obesity and the cutoffs of corresponding waist circumference (WC) and body mass index (BMI) in patients with Type 2 Diabetes (T2D). Cross-sectional cohort study. 1057 patients with T2D (550 males and 507 females) aged 18 or above that satisfied the criteria were included. The definition and diagnostic criteria of Metabolic syndrome (Mets) were analyzed according to the 2020 Chinese Diabetes Society (CDS) Guideline. The VFA and SFA were measured by bioelectrical impedance analysis (BIA). The optimal VFA and SFA cutoffs and corresponding WC and BMI when two or more nonadipose components of MetS (without central obesity) were met were analyzed by ROC curve. Among all of the T2D patients, the optimal VFA cutoff for identifying two or more nonadipose components of MetS was 73.30 cm^2^ for females and 69.20 cm^2^ for males, while the optimal SFA cutoff was 186.70 cm^2^ for females and 123.30 cm^2^ for males. The ROC area under curve (AUC) of VFA for identifying two or more nonadipose components of MetS was higher than that of SFA (Female: 0.65 vs. 0.58, *P* = 0.01). The VFA cutoff of newly diagnosed T2D patients (females = 86.10 cm^2^, males = 69.00 cm^2^) was higher than that of non-newly diagnosed T2D patients (females = 73.30 cm^2^, males = 65.40 cm^2^). A stratification analysis of gender and whether newly diagnosed with T2D or not showed that the WCs corresponding to VFA were 85.00 cm and BMI was about 24.00 kg/m^2^. VFA measured by BIA can be a non-invasive method to detect central obesity in patients with T2D, the corresponding WC were 85.00 cm and BMI was 24.00 kg/m^2^.

## Introduction

Metabolic syndrome (Mets) is a group of metabolically interrelated risk factors. Obesity, hyperglycemia, hypertension and dyslipidemia are core components of metabolic syndrome^1^. Metabolic syndrome seriously affects health, directly induces atherosclerotic cardiovascular disease (ASCVD) and increases the risk of T2D^2^. With the change of lifestyle in China, the prevalence of obesity and overweight is increasing year by year, directly pushing up the prevalence of metabolic syndrome. According to statistics, at present, the prevalence of metabolic syndrome in big cities in China is about 9–12%, but it varies across region, gender and age group^3^. The diagnostic criteria of metabolic syndrome vary from institution to institution, and it is mainly diagnosed by central obesity, blood glucose, blood lipid and blood pressure. So far, it is assumed that central obesity and insulin resistance are the two most important pathological mechanisms of metabolic syndrome, they facilitate each other and cause each other, and central obesity is the basis^3^. Central obesity, also known as centripetal, visceral or abdominal obesity, is defined as excessive visceral fat or abdominal fat. Studies have shown that the excess of visceral fat is more harmful than that of other parts of fat, so central obesity is accepted as one of the diagnostic criteria for metabolic syndrome^4^.

VFA is used as the diagnostic criterion for central obesity, but the VFA cutoff for diagnosing central obesity varies considerably across races and populations, and the corresponding waist circumferences (WC) also differ^5,6^. According to the diagnostic criteria of Chinese Diabetes Society (CDS), the VFA cutoff for diagnosing central obesity in Chinese population is 80.00 cm^2^, and the corresponding WC is ≥ 90.00 cm for males and ≥ 85.00 cm for females^7,8^. Compared with the general population, T2D patients are more likely to have such complications as blood lipid metabolism and hypertension. For this reason, we wonder whether the VFA cutoff and corresponding WC of central obesity applies to T2D patients in CDS’s guidelines for diagnosis of metabolic syndrome. Obesity and central obesity of general population in China refer to the cutoff of *Health Industrial Standard of the People's Republic of China* in 2013 (WS/T 428-2013), which was dated nearly a decade ago and explicitly explains that it is not applicable to particular population(i.e. T2D patients). Many T2D patients may present with central obesity and metabolic syndrome despite normal weight and BMI, but less aware of their condition. Long-term untreated and unmonitored central obesity can lead to the occurrence and development of related complications.

What’s more, few studies have discussed the use of VFA, body mass index (BMI) and WC as the optimal cutoffs of obesity in T2D patients. Therefore, our study took T2D patients as the research objects, and aimed to determine the cutoff values of various anthropometric indicators that can help diagnose central obesity.

CT and MRI are gold standards for evaluating VFA. Due to high technical cost and high radiation exposure, however, these techniques are restricted in clinical application and not applicable to the screening of large population. Previous studies have shown that the use of BIA can provide accurate measurements of VFA. The comparison of VFA measured by BIA and CT revealed a high correlation between the two, moreover, in contrast to CT, BIA offered the advantages of being non-invasive, radiation-free, allowing dynamic observation, easy to operate and less costly^9,10^. In this study, we intended to use BIA method to test VFA and SFA in T2D patients, determine an appropriate cutoff for identifying two or more nonadipose components of MetS but not including central obesity in T2D patients, and determine corresponding WC and BMI cutoffs, so as to offer a simple and pragmatic diagnostic cutoff for measurement index of central obesity in T2D patients.

## Methods

### Research method and population

By using a cross-sectional cohort study design, T2D inpatients aged 18 or above in the Department of Endocrinology, The Second Affiliated Hospital of Guangzhou Medical University from 2019 to 2021 were selected. Based on the statistical parameters specified in this study, including an AUC of approximately 0.7, a test power with a confidence level ≥ 0.9, the presence or absence of two or more nonadipose components in patients (No/Yes = 0.19), and a *P* value ≤ 0.05 indicating statistical significance, a minimum of 150 T2D patients is required. All the clinical data were extracted from the medical records. The exclusion criteria were: (1) receiving systemic corticosteroid therapy now; (2) with severe hepatic and renal insufficiency; (3) hyperthyroidism or hypothyroidism; (4) severe disability and mental disorder; (5) pregnancy; (6) missing the necessity data for this study. All comorbidities of the patients with type 2 diabetes were derived from the final diagnosis shown in the patients’ hospitalization records, and all of these diagnoses were confirmed by the signature of a competent physician with a professional title of attending physician or above. The present study had been approved by the Ethics Committee of the Second Affiliated Hospital of Guangzhou Medical University (Approval No.: 2020-hs-29). In order to protect the privacy of patients, we do not collect the sensitive individual information such as name, family address, etc.

Chronic kidney disease (CKD) was defined as eGFR < 60 ml/min per 1.73 m^2^ and/or proteinuria > 300 mg/dl. The newly diagnosed T2D was defined as the patients was diagnosed as T2D for the first time and has never been treated with hypoglycemic drugs. Current smoking was defined as having smoked at least 100 cigarettes in one’s life and currently smoking cigarettes. Drinking history refers to at least once a month, including entertainment, or drinking more than half a year, that is, with drinking history; Educational status refers to the educational background is divided into primary school and below, secondary school and junior college or above. Fatty liver should be diagnosed by means of abdominal ultrasound. The hypoglycemic regimen was divided into oral hypoglycemic drugs, injection of insulin/GLP-1AR and oral hypoglycemic drugs + injection of insulin/GLP-1AR. The study was approved by the Ethics Committee of the Second Affiliated Hospital of Guangzhou Medical University and conducted in accordance with the ethical principles of the Declaration of Helsinki. Because of the retrospective study design, the Ethics Committee of the Second Affiliated Hospital of Guangzhou Medical University waived with the requirement of informed consent.

### Determination of body fat

Body fat, height (CM), weight (kg) and other anthropometric indicators were measured as per standard. The calculation method of body mass index (BMI) was the weight (kg) divided by the square of height (CM). WC was measured on the horizontal plane between the lower margin of ribs and the iliac bone on the midaxillary line. Hip circumference refers to the horizontal length of the posterior most prominent part of the buttocks.

VFA and SFA were measured in patients on an empty stomach by specialized full-time nurses with an Omron visceral fat analyzer (DUALSCAN HDS-2000, Japan): the examinees were told to start fasting at 20:00 the day before examination; and instructed to lie flat in supine position, expose their ankles, wrists and abdominal skin, and breathe calmly. At the end of calm exhalation, the patients were told to hold breath. At this point, the cross-sectional area of abdomen flush with navel was measured. Then an abdominal electrode belt and hand and foot electrode clamps were installed and the examinees were instructed to breathe calmly. At the end of calm exhalation, the patients were told to hold breath. At this point, the abdominal VFA and SFA were measured.

### Serological test

After fasting for 8–10 h, blood samples were collected from all of the patients in the early morning the day following admission to measure fasting blood glucose (FPG), fasting C-peptide (FCP), glycosylated hemoglobin (HbA1c), total cholesterol (TC), triglyceride (TG), high density lipoprotein cholesterol (HDL-C), low density lipoprotein cholesterol (LDL-C), uric acid (UA) and serum creatinine (Scr). The levels of 2-h postprandial C-peptide (2hCP) were determined in all subjects. The FPG were measured by glucose oxidase method, FCP and 2hCP were measured by chemiluminescence immunoassay (Cobas e 602, Roche, Switzerland), blood lipid was measured by C8K automatic analyzer (cobas8000, Roche, Switzerland) and HbA1c was determined by automatic erythrocyte sedimentation rate analyzer (LABNOVATION LD-600, LABNOVATION, China).

### Definition of metabolic syndrome

According to the 2020 CDS Guidelines, the diagnostic criteria of Mets were as follows: a definite diagnosis can be made if three or more of the following items were met^7^. (1) Central obesity (abdominal obesity): WC ≥ 90.00 cm for males and ≥ 85.00 cm for females. (2) Hyperglycemia: fasting blood glucose ≥ 6.10 mmol/L or blood glucose ≥ 7.80 mmol/L 2 h after glycemic load and/or those who were diagnosed with diabetes and treated. (3) Hypertension: blood pressure ≥ 130/85 mmHg (1 mmHg = 0.133 kPa) and/or those who were identified as hypertensive and treated. (4) Fasting triglyceride (TG) ≥ 1.70 mmol/L. (5) Fasting HDL‐C < 1.04 mmol/L. According to the above diagnostic criteria, except for central obesity, those who met two or more of the above criteria were defined as two or more nonadipose components.

### Statistical analysis

**Figure 1:**
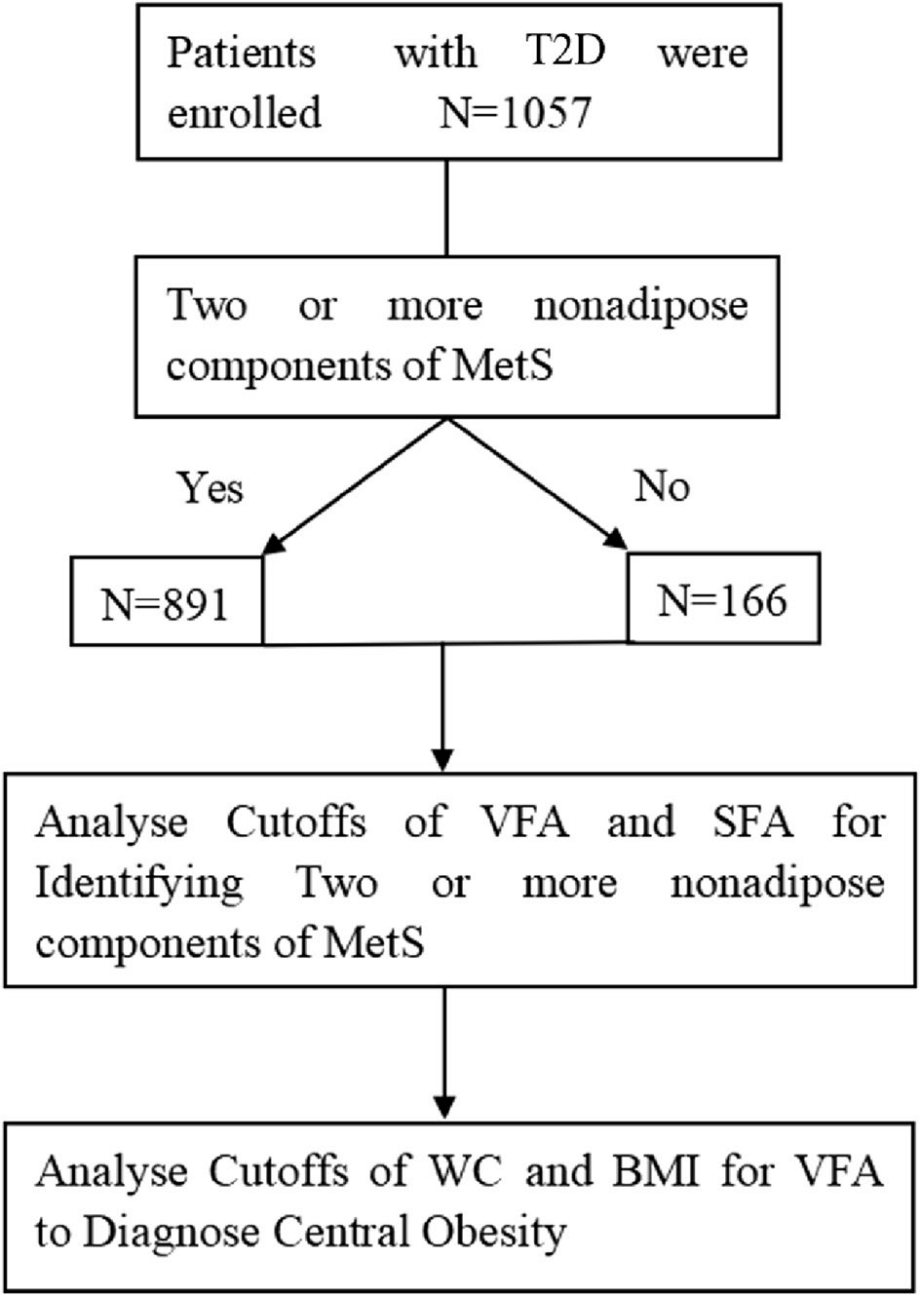
Flowchart of this research.

Continuous variables in normal distribution were expressed as mean ± SD, and t-test was adopted for inter-group comparison. Continuous variables not in normal distribution were expressed as median (quartile range: 25–75%), and Wilcoxon rank sum test was adopted for inter-group comparison. Enumeration data or categorical data were expressed as frequency or percentage (%), and chi-square test was employed for inter-group comparison. VFA and SFA cutoffs were evaluated by ROC curve analysis when two or more Mets diagnostic indicators were met in the absence of diagnostic conditions for central obesity. After that, the corresponding cutoffs of WC, BMI and other body measurement indexes were evaluated using the same method, with *P* ≤ 0.05 indicating that the difference was statistically significant. Intra-assay and inter-assay CV for each method was calculated as: CV = (SD/Mean) × 100%. Empower (R) (www.empowerstats.com, X&Y Solutions, Inc Boston, MA) and R (http://www.r-project.org) were used for all statistical analyses in this study. The flow chart of this research is shown in Fig. 1.

### Ethics approval

The present study had been approved by the Ethics Committee of the Second Affiliated Hospital of Guangzhou Medical University (Approval No.: 2020-hs-29).

## Results

### General clinical features of T2D patients

A total of 1057 T2D patients were included in this study, of whom 891 patients satisfied two or more nonadipose components of MetS defined by CDS, and the proportion of males was higher than that of females (females = 416, 46.69%; males = 475, 53.31%, *P* = 0.05). Regardless of gender, patients who met two or more nonadipose components of MetS had higher VFA, SFA, WC, BMI and age than those who didn’t meet two nonadipose components of MetS, higher incidences of hyperuricemia, renal insufficiency, peripheral neuropathy, cerebral infarction and coronary disease, as well as higher fasting and 2-h postprandial C-peptide (Table 1).

**Table 1:** General clinical features of T2D patients.

	Two or more nonadipose components (without WC) by the CDS definition		*P*-value
	No	Yes	
Number of cases (N)	166	891	
Age (years)	58.70 ± 12.42	61.09 ± 14.41	0.04
BMI (kg/m^2^)	23.74 ± 3.82	25.37 ± 4.21	< 0.001
WC (cm)	82.95 ± 8.44	88.35 ± 9.79	< 0.001
Hip circumference (cm)	92.56 ± 8.59	94.74 ± 8.57	0.005
Visceral fat area (VFA, cm^2^)	70.03 ± 37.75	90.45 ± 42.34	< 0.001
Subcutaneous fat area (SFA, cm^2^)	160.29 ± 68.13	186.92 ± 77.42	< 0.001
Fasting blood glucose (mmol/L)	7.97 ± 3.44	8.00 ± 3.69	0.94
Fasting C-peptide (μg/L)	1.74 ± 1.11	2.62 ± 1.64	< 0.001
2-h postprandial C-peptide (μg/L)	4.08 ± 3.43	4.85 ± 3.18	< 0.001
HbA1c (%)	9.90 ± 2.81	9.71 ± 2.77	0.41
Total cholesterol TC (mmol/L)	4.81 ± 1.07	4.75 ± 1.39	0.584
Triglyceride TG (mmol/L)	1.05 ± 0.29	2.15 ± 2.17	< 0.001
HDL-C (mmol/L)	1.40 ± 1.05	0.98 ± 0.26	< 0.001
LDL-C (mmol/L)	3.20 ± 1.03	3.03 ± 1.16	0.09
C-peptide insulin resistance index	1.51 ± 0.00	1.51 ± 0.01	< 0.001
C-peptide insulin sensitivity index	0.08 ± 1.28	0.30 ± 1.94	< 0.001
Gender			
Female	91 (54.80%)	416 (46.70%)	0.05
Male	75 (45.20%)	475 (53.30%)	
Smoking history			
No	142 (85.50%)	753 (84.50%)	0.73
Yes	24 (14.50%)	138 (15.50%)	
Drinking history			
No	158 (95.20%)	821 (92.10%)	0.16
Yes	8 (4.83%)	70 (7.9%)	
Education background			
Primary school and below	51 (30.70%)	237 (26.60%)	0.36
Secondary school	82 (49.40%)	493 (55.30%)	
Junior college or above	33 (19.90%)	161 (18.10%)	
Family history			
No	126 (75.90%)	666 (74.80%)	0.75
Yes	40 (24.10%)	225 (25.30%)	
Diabetic foot			
No	163 (98.20%)	860 (96.50%)	0.26
Yes	3 (1.80%)	31 (3.50%)	
Newly diagnosed T2D			
No	114 (68.70%)	663 (74.40%)	0.12
Yes	52 (31.30%)	228 (25.60%)	
With CKD			
No	146 (88.00%)	618 (69.40%)	< 0.001
Yes	20 (12.00%)	273 (30.60%)	
With retinopathy			
No	158 (95.20%)	821 (92.10%)	0.17
Yes	8 (4.80%)	70 (7.90%)	
Coronary disease			
No	160 (96.40%)	766 (86.00%)	< 0.001
Yes	6 (3.60%)	125 (14.00%)	
With peripheral vascular disease			
No	110 (66.30%)	503 (56.50%)	0.02
Yes	56 (33.70%)	388 (43.50%)	
Cerebral infarction			
No	144 (86.80%)	699 (78.50%)	0.02
Yes	22 (13.20%)	192 (21.50%)	
With peripheral neuropathy			
No	99 (59.60%)	560 (62.90%)	0.43
Yes	67 (40.40%)	331 (37.10%)	
Hyperuricemia			
No	154 (92.80%)	711 (79.80%)	< 0.001
Yes	12 (7.20%)	180 (20.20%)	
With hypertension			
No	166 (100%)	319 (35.80%)	< 0.001
Yes	0 (0%)	572 (64.20%)	
Fatty liver			
No	119 (71.69%)	518 (58.14%)	0.001
Yes	47 (28.31%)	373 (41.86%)	
Hypoglycemic regimen			
Oral hypoglycemic drugs	40 (24.10%)	198 (22.40%)	0.77
Injection of insulin/GLP-1RA	70 (42.17%)	399 (45.14%)	
Oral hypoglycemic drugs + Injection of insulin/GLP-1RA	56 (33.73%)	287 (32.47%)	

Results in this table: mean + SD/N (%); if it was a continuous variable, it was obtained by Kruskal Wallis rank sum test. If the counting variable had a theoretical number < 10, it was obtained by Fisher’s exact test.

### Optimal cutoffs of VFA and SFA and Various parameters for identifying two or more nonadipose components of MetS

**Figure 2:**
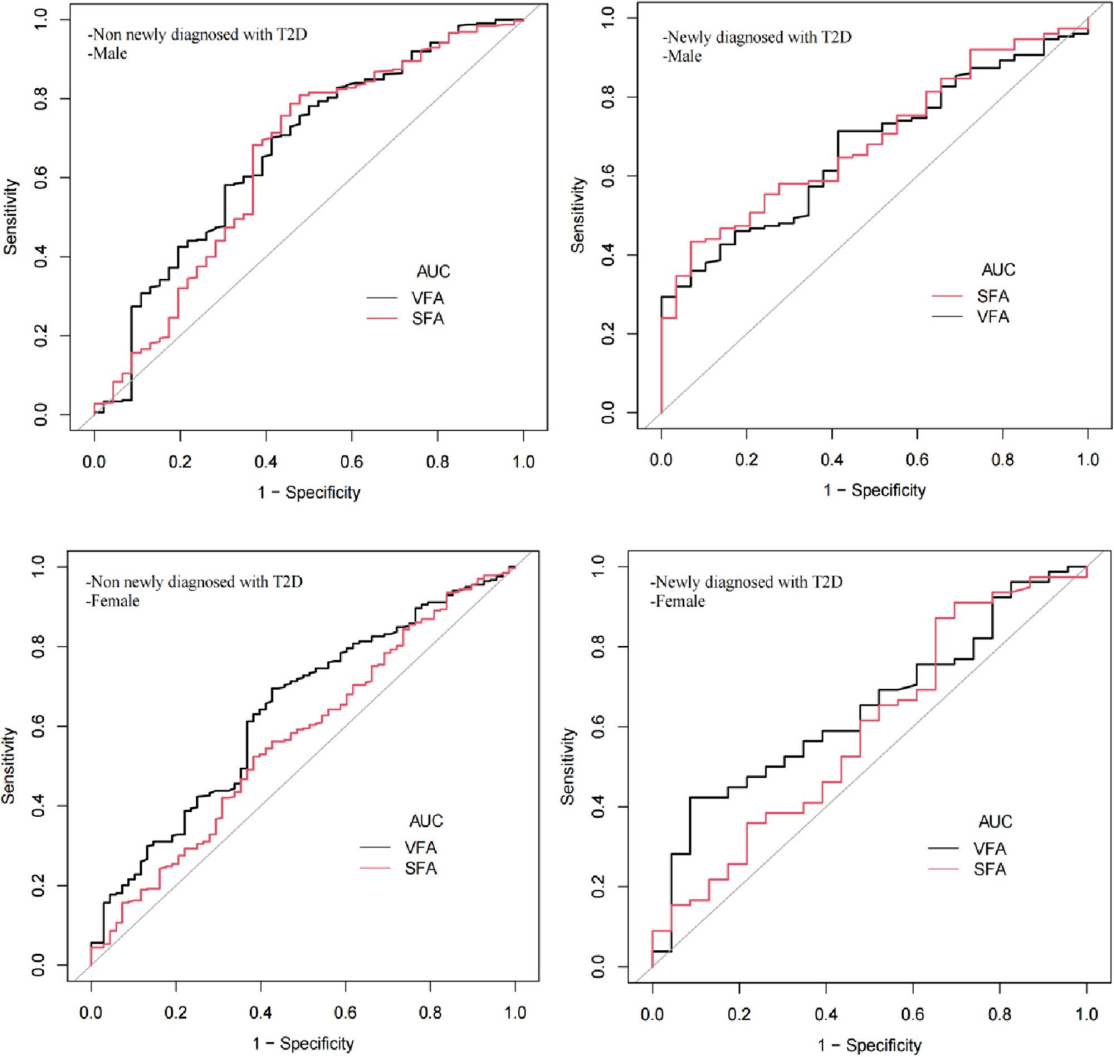
The ROC curves for VFA and SFA to predict the presence of two or more nonadipose components of MetS, which were classified into male new diagnosis, female new diagnosis, male non-new diagnosis and female non-new diagnosis.

Table 2 and Fig. 2 gives a detailed list of specific values of VFA and SFA for identifying two or more nonadipose components of MetS, including sensitivity, specificity, positive likelihood ratio, negative likelihood ratio and diagnostic accuracy. In the whole T2D population, based on the CDS definition, the optimal cutoff for different measurement indexes to identify two or more nonadipose components of MetS was: in male patients, VFA was 69.20 cm^2^ and SFA was 186.70 cm^2^. The ROC AUCs of VFA and SFA were 0.67 and 0.66, respectively, and there was no statistical difference between them in AUC. In female patients, VFA was 73.40 cm^2^ and SFA was 186.70 cm^2^, and the ROC AUC of VFA was 0.65, higher than that of SFA (AUC = 0.58, *P* = 0.01). We then performed a subgroup stratification analysis according to whether T2D patients were newly diagnosed or not and the results indicated that the diagnostic VFA cutoff (86.20 cm^2^) in newly diagnosed female T2D patients was higher than that in non-newly diagnosed patients (73.40 cm^2^), but SFA was on the contrary (186.70 cm^2^ vs. 111.00 cm^2^). The diagnostic cutoffs of VFA and SFA in newly diagnosed male T2D patients were higher than those in non-newly diagnosed patients (see Table 2 for details).

**Table 2:** Various parameters for VFA and SFA to identify two or more nonadipose components of MetS.

	AUC (95% CI)	P value for AUC vs*.* VFA	Best cutoff	Specificity	Sensitivity	Accuracy	Postive-pv	Negative-pv
Gender = F								
Total								
VFA	0.65 (0.59–0.71)		73.40	0.61	0.65	0.65	0.89	0.28
SFA	0.58 (0.52–0.65)	0.01	186.70	0.65	0.48	0.52	0.87	0.22
Newly diagnosed with T2D								
VFA	0.65 (0.52–0.77)		86.20	0.91	0.42	0.53	0.94	0.32
SFA	0.59 (0.45–0.73)	0.24	111.00	0.34	0.87	0.75	0.82	0.44
Not newly diagnosed with T2D								
VFA	0.64 (0.56–0.71)		73.40	0.57	0.70	0.68	0.89	0.27
SFA	0.57 (0.49–0.65)	0.03	186.70	0.62	0.52	0.54	0.87	0.21
Gender = M								
Total								
VFA	0.67 (0.60–0.73)		69.20	0.60	0.67	0.66	0.91	0.22
SFA	0.66 (0.60–0.73)	0.89	123.20	0.46	0.80	0.76	0.91	0.27
Newly diagnosed with T2D								
VFA	0.67 (0.58–0.76)		69.00	0.59	0.71	0.69	0.90	0.28
SFA	0.69 (0.60–0.78)	0.65	185.50	0.93	0.43	0.51	0.97	0.24
Not newly diagnosed with T2D								
VFA	0.67 (0.58–0.76)		65.40	0.587	0.701	0.69	0.92	0.22
SFA	0.65 (0.55–0.74)	0.40	125.40	0.543	0.787	0.76	0.92	0.27

*Positive-pv* positive predictive value, *Negative-pv* negative predictive value.

### Optimal WC and BMI cutoffs for VFA value to diagnose central obesity

According to the previous findings, the above VFA cutoffs were taken as the diagnostic cutoffs of central obesity, and the corresponding WC and BMI cutoffs were selected using the area of ROC curve. According to the CDS definition, in female T2D patients, when VFA = 73.40 cm^2^ was selected to diagnose central obesity, the corresponding WC was 84.50 cm and BMI was 23.80 kg/m^2^. In male T2D patients, when VFA = 69.20 cm^2^ was selected to diagnose central obesity, the corresponding WC was 85.50 cm and BMI was 24.73 kg/m^2^. Then a subgroup stratification analysis was performed according to whether T2D patients were newly diagnosed or not and the corresponding VFA cutoffs were analyzed. The results indicated that whether for newly diagnosed or non-newly diagnosed female or male T2D patients, the corresponding WC and BMI didn’t differ a lot. The WCs of four subgroups were 85.00 cm, and the BMI was 24.00 kg/m^2^ or so. See Table 3 for details.

**Table 3:** Optimal WC and BMI cutoffs and relevant parameters for VFA value to diagnose central obesity.

	VFA	ROC area	Best cutoff	Specificity	Sensitivity	Accuracy	Postive-pv	Negative-pv
Gender = F								
Total	73.40							
WC (cm)		0.88	84.50	0.83	0.75	0.78	4.46	0.29
BMI (kg/m^2^)		0.86	23.80	0.76	0.84	0.81	3.56	0.20
Newly diagnosed with T2D	86.20							
WC (cm)		0.90	83.30	0.82	0.85	0.83	4.65	0.18
BMI (kg/m^2^)		0.89	23.90	0.82	0.89	0.84	4.87	0.13
Not newly diagnosed with T2D	73.40							
WC (cm)		0.87	85.50	0.85	0.72	0.76	4.94	0.33
BMI (kg/m^2^)		0.85	24.30	0.80	0.79	0.79	3.98	0.26
Gender = M								
Total	69.20							
WC (cm)		0.87	85.50	0.78	0.81	0.80	3.60	0.24
BMI (kg/m^2^)		0.79	24.70	0.86	0.60	0.69	4.19	0.46
Newly diagnosed with T2D	69.00							
WC (cm)		0.85	85.50	0.75	0.81	0.79	3.16	0.26
BMI (kg/m^2^)		0.77	24.80	0.80	0.60	0.66	2.98	0.50
Not newly diagnosed with T2D	65.40							
WC (cm)		0.89	85.50	0.79	0.82	0.81	4.01	0.22
BMI (kg/m^2^)		0.81	23.80	0.79	0.73	0.75	3.48	0.33

*Positive-pv* positive predictive value, *Negative-pv* negative predictive value.

## Discussion

Overweight and obesity have become the most common metabolic disorders in the world. Nowadays, the prevalence of obesity (BMI > 30.00 kg/m^2^) in the world is estimated to exceed 30%^11^. Central obesity means that fat is mainly deposited subcutaneously and intraperitoneally, and it is also a high-risk factor for metabolic syndrome, diabetes and cardio-cerebrovascular diseases (CCDs)^12^. VFA is taken as a diagnostic gold standard of central obesity. VFA cutoffs can be influenced by a variety of factors, including age, gender, ethnic group and presence of other complications. The VFA cutoff of central obesity in Japanese population is greater than 100.00 cm^2^^13^, similar to that in Korean population, that is, 103.00 cm^2^^14^. In the Chinese population, according to the metabolic syndrome criteria by the CDS definition, the optimal VFA cutoffs for diagnosing male and female central obesity are 80.00 cm^2^^7,8^. So far, the VFA cutoff for diagnosing central obesity in T2D patients hasn’t been determined. In our work, T2D was taken as the research object, and BIA was adopted to detect VFA and SFA. It was found that the optimal VFA cutoff for diagnosing central obesity was 73.40 cm^2^ for females and 69.20 cm^2^ for males. And the SFA cutoff was 186.70 cm^2^ for females and 69.20 cm^2^ for males. The ROC AUC of VFA for identifying two or more nonadipose components of MetS was higher than that of SFA. The VFA cutoffs in newly diagnosed T2D patients (female = 86.10 cm^2^, male = 69.00 cm^2^) were higher than those in non-newly diagnosed T2D patients (female = 73.30 cm^2^, male = 65.40 cm^2^). Our results were consistent with previous studies. VFA can better reflect metabolic disorder and is more effective in diagnosing central obesity than SFA^15^. But the VFA cutoff for diagnosing central obesity detected by BIA method in this study was lower than that in the previous surveyed population, probably because diabetic patients were prone to a series of metabolic disorders, such as dyslipidemia, hypertension and even hyperuricemia. Consequently, the VFA cutoff that can identify the components of metabolic syndrome in diabetic population was lower than that in general population. We performed a subgroup analysis according to whether newly diagnosed with diabetes or not, the VFA cutoffs for diagnosing central obesity in newly diagnosed male and female patients were greater than those in non-newly diagnosed patients. Due to the longer course of disease and relatively older age of non-newly diagnosed patients, they were at a higher risk of blood lipid metabolism disorder or hypertension. This may be an important reason why their VFA cutoff for diagnosing central obesity was lower.

This coincided with the above speculation. Some studies have also found that the VFA detected by BIA method was lower than those detected by CT or MRI methods in the diagnosis of central obesity, which may be one of the reasons why the VFA was lower than the previously reported value, that is, 80.00 cm^2^, in the diagnosis of central obesity in T2D patients^16^. In future studies, BIA method should be adopted to detect VFA in the general population, and a standard VFA cutoff detected by BIA method for diagnosing central obesity should be established to determine the accuracy and reproducibility of VFA detected by BIA method.

BIA solves the problems of high cost and radiation of CT or MRI in detecting VFA and is widely used in clinical and epidemiological studies^17^. However, the cost of BIA remains high, and can’t come into common use in different medical institutions at all levels in China. The detection of VFA requires a simple, universal and highly usable alternative anthropometric indicator. Waist circumference (WC) is still the most ideal index to replace VFA in diagnosing central obesity^6^. In this work, the optimal WC and BMI were selected based on area under the curve (AUC), so as to determine the optimal VFA cutoff measured by BIA. The results indicated that when VFA = 73.30 cm^2^ was selected to diagnose central obesity in female T2D patients, the corresponding WC was 84.50 cm and BMI was 23.70 kg/m^2^. When the VFA of male patients was 69.20 cm^2^, the corresponding WC was 85.50 cm and BMI was 24.70 kg/m^2^. In the subgroup analysis of whether newly diagnosed with diabetes or not, there was little difference between male and female patients in WC and BMI for diagnosing central obesity, all WCs were 85.00 cm and BMIs were about 24.00 kg/m^2^. The VFA values in different groups in the research results show little difference in the corresponding cutoff values of WC and BMI, especially that of BMI. Similar findings have been reported in previous studies^8^, suggesting that BMI has certain limitations in determining central obesity. For VFA values for diagnosing central obesity in China, the corresponding WC was greater than 90.00 cm for males and greater than 85.00 cm for females. They were values with the widest applicability in the general population in China^7^. Affected by the measurement method, choice of surveyed population and other factors, the VFA for diagnosing central obesity in T2D patients in this study was lower than the previously reported cutoff, and the corresponding BMI and WC also differed. The existing BMI and WC cutoffs for diagnosing obesity in China may underestimate the obesity degree of T2D patients in China. However, some studies have suggested taking BMI ≥ 25.00 kg/m^2^ should be regarded as obesity, WC ≥ 93.00 cm in males and WC ≥ 90.00 cm in females are optimal cutoffs for diagnosing central obesity in T2D, probably because different studies have different definitions and measurement methods for obesity or central obesity, and the corresponding WC and BMI cutoffs differ accordingly. The race is also an important factor affecting the optimal WC cutoff for diagnosing obesity. According to reported statistics, in different study populations, the WC values for diagnosing central obesity was 65.50–101.20 cm for females and 72.50–103.00 cm for males^19–22^, and the WC value for diagnosing central obesity reported by us was within this range. In China, the VFA cutoff and WC for diagnosing central obesity in males are generally higher than those in females. But our results revealed that the WC for diagnosing central obesity in male T2D patients was equal to that of female patients, probably because male patients had a higher smoking rate and drinking rate, coupled with the risk factor of hyperglycemia. So they were prone to metabolic problems, such as blood pressure and blood lipid when VFA and WC cutoffs were low. Research has found that a majority of T2D patients have dyslipidemia, also known as diabetic dyslipidemia, characterized by decreased HDL and elevated LDL and triglycerides. These abnormal lipid components can manifest as either single or mixed dyslipidemia. Despite a combination of intervention strategies, such as drugs or lifestyle modification, T2D patients still face a high risk of cardiovascular adverse events (CVD). The utilization of simple and practical body anthropometric indicators can help T2D patients self-monitor their central obesity and control complications related to dyslipidemia^23–25^.

Our work was targeted at a surveyed population collected from a single hospital in Guangzhou City, Guangdong Province, China^26^. Our research objects mainly came from southern China, and different regions had different lifestyles and living standards. Thus, our results can’t represent the whole population, and it remained uncertain whether the research conclusions were applicable to other populations. Further studies were needed in other parts of China, to determine the diagnostic VFA and corresponding WC and BMI cutoffs in T2D patients. In this work, VFA was measured by BIA. Although this method had a strong correlation with CT and MRI, its accuracy in calculating VFA was lower than CT and MRI and was not the gold standard for measuring VFA. Moreover, it was also susceptible to changes in body fluid, such as water intake, diet, diarrhea, and exercise, and these conditions should be taken into account when measuring VFA.

In addition, the error among BIA instruments was also worthy of our attention, so as to avoid the data differences caused by heterogeneity among different detection methods and detection instruments. Secondly, this study was designed as a cross-sectional study and can’t explain the causality between VFA size and Mets. Prospective cohort study was a necessary option to explain the relationship between VFA and Mets. We selected Mets as the end point of study and observation, and obtained corresponding VFA, WC and BMI values. For diabetic patients, however, the future research focus may be selecting diabetes-related complications, such as renal insufficiency and cardiovascular complications, etc. as the research outcome to determine the optimal predictive cutoffs for VFA and WC.

To sum up, this study documents that the VFA values measured by BIA for diagnosing central obesity in T2D patients are 70.00 cm^2^ for males and 73.00 cm^2^ for females. However, we should pay attention to whether newly diagnosed with T2D or not and make adjustment accordingly. The WC cutoff corresponding to VFA cutoff in this study is 85 cm and BMI is 24.00 kg/m^2^, which may be a simple and usable measurement index for central obesity in T2D patients in clinical and epidemiological studies.

## Data Availability

The datasets used and/or analysed during the current study are available from the corresponding author on reasonable request.
